# Abnormal Auditory Brainstem Response (ABR) Findings in a Near-Normal Hearing Child with Noonan Syndrome

**Published:** 2017-01

**Authors:** Bahram Jalaei, Mohd Normani Zakaria, Dinsuhaimi Sidek

**Affiliations:** 1 *Department of Audiology, Faculty of Rehabilitation, Iran University of Medical Sciences, Tehran, Iran.*; 2 *Audiology Program, School of Health Sciences, University Sains Malaysia, Kelantan, Malaysia.*; 3 *Department of Otorhinolaryngology, School of Medical Sciences, University Sains Malaysia, Kelantan, Malaysia.*

**Keywords:** Audiological evaluation, Auditory Brainstem response (ABR), Hearing loss, Noonan Syndrome

## Abstract

**Introduction:**

Noonan syndrome (NS) is a heterogeneous genetic disease that affects many parts of the body. It was named after Dr. Jacqueline Anne Noonan, a paediatric cardiologist.

**Case Report:**

We report audiological tests and auditory brainstem response (ABR) findings in a 5-year old Malay boy with NS. Despite showing the marked signs of NS, the child could only produce a few meaningful words. Audiological tests found him to have bilateral mild conductive hearing loss at low frequencies. In ABR testing, despite having good waveform morphology, the results were atypical. Absolute latency of wave V was normal but interpeak latencies of wave’s I-V, I-II, II-III were prolonged. Interestingly, interpeak latency of waves III-V was abnormally shorter.

**Conclusion:**

Abnormal ABR results are possibly due to abnormal anatomical condition of brainstem and might contribute to speech delay.

## Introduction

Noonan syndrome (NS) is a heterogeneous genetic disease that affects many parts of the body. It was named after Dr. Jacqueline Anne Noonan, a paediatric cardiologist, who in year 1962 identified 9 patients with faces that were remarkably similar and had short stature, skeletal malformations, significant chest deformities, and pulmonary stenosis. This condition occurred in both genders, was associated with normal chromosomes, included congenital heart defects and could be familial (1). NS is present in about 1 in 1,000 to 1 in 2,500 people (2).

People with NS have distinctive facial features such as a deep groove in the area between the nose and mouth (philtrum), widely spaced eyes that are usually pale blue or blue-green in color, and low-set ears that are rotated backward. Affected individuals may have a high arch in the roof of the mouth (high-arched palate), poor alignment of the teeth, and a small lower jaw. Many children with NS have a short neck and both children and adults may have excess neck skin (also called webbing) and a low hairline at the back of the neck. Approximately 50 to 70 percent of individuals with NS have short statures (3-5). At birth, they are usually of normal length and weight, but the growth slows over time. 

The presence of hearing loss in NS has also been reported. Sharland et al. reported abnormal hearing in 40% of 151 patients who had NS (3). Heller reported bilateral nerve deafness in one case of NS (6). Duenas et al. reported hearing conduction impairment in three out of 25 cases (7). Meanwhile, Cremers et al. reported perceptive hearing loss and mental retardation in two patients with NS (8). Miura et al. conducted a histopathological study on the left temporal bones of 3 subjects after their death (9). They indicated that the cause of conductive and sensorineural hearing loss could be related to mesenchymal tissue in the middle ear and endolymphatic hydrops in the inner ear, respectively. In addition, they also reported abnormalities in the inner ear and central auditory nervous system (CANS) such as shortening of the cochlea and reduced spiral ganglion cells, respectively. A more recent study found that a great percentage of NS subjects had external ear anomalies in addition to conductive and sensorineural hearing losses (10). The hearing aspect of patients with NS has been studied to some extent. Based on the literature, subjects with NS might have sensorineural hearing loss which could be due to cochlear or retro-cochlear dysfunction. To the best of the authors’ knowledge, no report has been published to investigate the auditory brainstem’s function in NS. Herein, we report basic audiological evaluations and auditory brainstem response (ABR) outcomes in a Malay boy with NS. 

## Case Report

**Fig 1:**
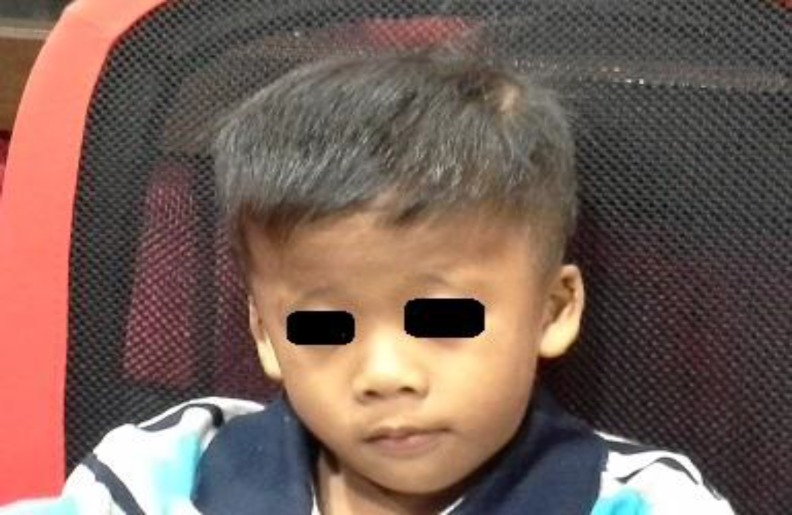
Facial characteristics of the 5-year old Malay boy with Noonan syndrome

**Fig 2:**
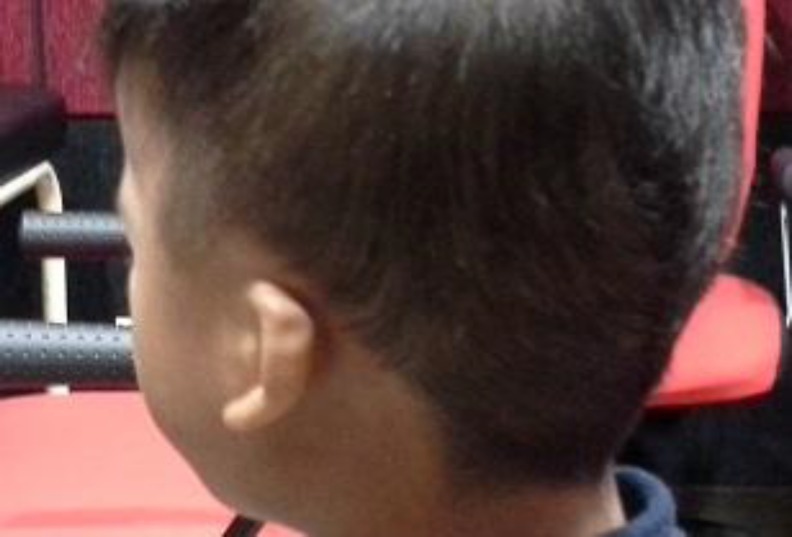
The view of left pinnae of the NS child

**Fig 3:**
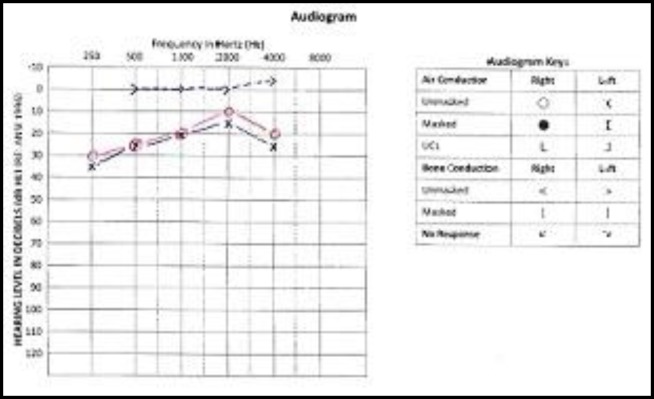
Play audiometry result of the NS child

**Fig 4:**
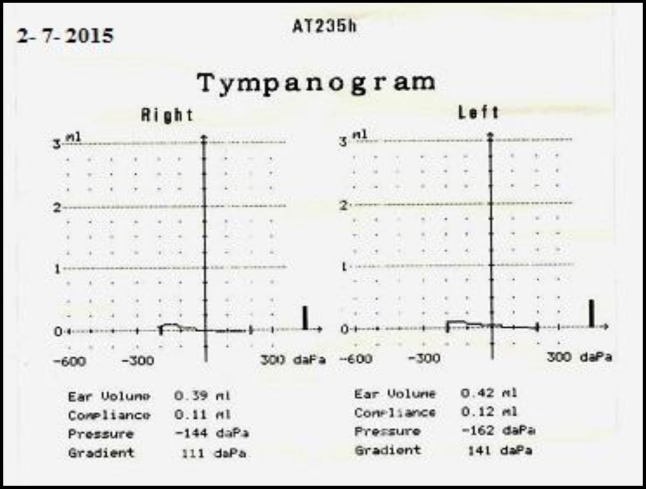
Tympanometric results for the NS child

This is a case of a 5-year old Malay boy who was born on June 24, 2009 (Fig. 1). According to the report of the genetic department of Hospital UniversitiSains Malaysia (HUSM), he is apparently a normal male karyotype 46, XY (25) but was diagnosed clinically as NS at the age of 18 months. According to the report of the paediatric clinic, he has soft dimorphism including low hair line, hypertelorism, down slanting palpebral fissures, bilateral epicanthic folds, and a deformed upper part of both pinnas (Fig.2). He was able to walk at 16 months and run at 17 months. Around this age, he was also able to climb stairs with help. His receptive and expressive abilities are unsatisfactory. Currently, at the age of 5 years, he can only understand simple commands. In terms of expressive language, a great delay was reported where he could only pronounce a few meaningful words. No evidence of mental retardation and expressive aphasia was reported. The patient visited the audiology clinic, HUSM, several times for a hearing assessment. The authors invited the patient for his latest visit to the audiology clinic in the faculty of Health of USM (July 2, 2015) and after written informed consent by his parents, otoscopic examination revealed an intact but dull tympanic membrane, bilaterally. Play audiometry was performed with a GSI 61 audiometer (Grason-Stadler Inc., USA) and it was observed that the patient had bilateral mild conductive hearing loss (CHL) that is more prominent at low frequencies (Fig.3). To assess middle ear function, tympanometry (AT235 model by Interacoustics A/S, Denmark) was carried out. Fig.4 shows the tympanometric results. As indicated, type B tympanogram (with a rounded peak) was obtained in both ears. Acoustic reflex testing was conducted and no reflexes were recorded at the tested frequencies (0.5, 1 and 2 kHz). An OtoReadTMportable device(Interacoustics A/S, Denmark) was used to record distortion product otoacoustic emission (DPOAE). Good outer hair cell emissions were noted at high frequencies (2 to 5 kHz) in both ears.

ABR was then performed to determine the brainstem status of the child. A two-channel Biologic Navigator Pro system (Natus Medical Inc., Mundelein, USA) was used to record ABR to clicks. Four scalp electrodes were placed on the child’s head: non-inverting on the vertex, inverting on each mastoid, and ground on the forehead. The electrodes impedance was kept below 3 kΩ throughout the testing.

Before the testing began, proper instructions were given to the child. After placing the headphones on his head, the stimulus was administered to each ear monaurally at 75 dBnHL. The stimulus rate was set at 10.3/s with 2000 sweeps. The epoch time was set at 15 ms (including a 1.21 ms pre-stimulus period). The acquired responses were amplified 100,000 times and band-pass filtered at 100-3000 Hz. To ensure good waveform replicability, the recording was repeated at least twice for each trial. During the testing, the subjects lied comfortably on the provided bed in the sound proof room of the audiology clinic in the HUSM.

**Fig 5:**
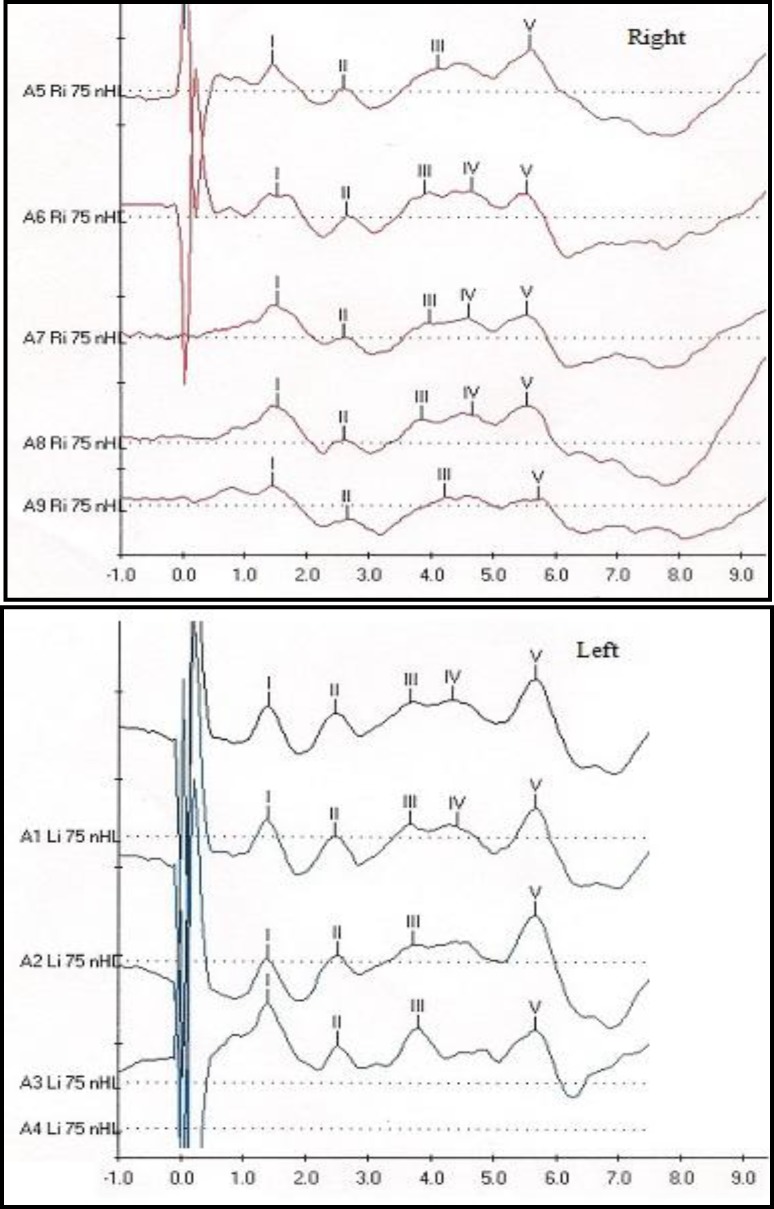
ABR results for the NS child

Fig.5 shows the ABR results for the NS child. The ABR morphology was unremarkable in both the left and right sides, as well as for different stimulus polarities. The values of ABR absolute latency and interpeak latencies (IPLs) are revealed in Table 1.

**Table 1:** Absolute latency and interpeak latencies of ABR peaks for the NS child and the respective normal child (in brackets) in the left and right ears

Ear	Polarity	Absolute latency V(ms)	Interpeak latency I-V (ms)	Interpeak latency I- III (ms)		Interpeak latency III- V (ms)
				I- II (ms)	II-III (ms)	
**Right**	Rarefaction	5.60 (5.59)	4.13 (3.98)	1.13 (0.96)	1.50 (1.13)	1.50 (1.89)
	Condensation	5.54	4.00	1.13	1.25	1.62
	Alternating	5.54 (5.51)	4.00 (3.85)	1.06 (0.94)	1.38 (1.08)	1.56 (1.83)
**Left**	Rarefaction	5.67 (5.64)	4.25 (3.93)	1.06 (0.9)	1.19 (1.12)	2.00 (1.91)
	Condensation	5.67	4.25	1.13	1.25	1.87
	Alternating	5.60 (5.58)	4.19 (3.86)	1.12 (0.96)	1.38 (1.06)	1.69 (1.84)

For comparison, the ABR results of a normal age-equivalent child are also provided (for this child, only the ABR data for rarefaction and alternating clicks are shown). As indicated, the absolute latency of wave V for the NS child was essentially similar to the normal child in both ears. The IPLs of I-V, I-II slightly and II-III were markedly prolonged in the NS patient compared to the normal child. Interestingly, the NS child revealed a much shorter IPL of III-V compared to that of the normal child in both ears. For example, in the right ear, for rarefaction clicks stimulation, the IPL of III-V were 1.50 ms and 1.89 ms for the NS child and the normal child, respectively. In addition, a good diagnostic value of alternating clicks in the ABR recording was also revealed. As shown in Table 1, for an IPL of III-V, the alternating clicks stimulation showed more consistent results than that of rarefaction clicks in both ears. On the contrary, the rarefaction clicks results were different for each ear (i.e. in the right side, a shorter IPL of III-V was noted in the NS child compared to the normal child and the results were opposite in the left ear). 

Based on routine audiological tests, there was a clear evidence that the child had conductive hearing loss. He was then referred to an otorhinolaryngologist for medical treatment. Due to the remarkable results in ABR, he was also referred for head imaging to verify the findings of ABR. He has been having regular visits to a speech pathology clinic to improve his speech and language abilities.

## Discussion

This case is of interest because, despite having mild hearing loss, the patient has a delay in speech. Play audiometry found him to have mild CHL at low frequencies bilaterally, which is consistent with the results of tympanometry, acoustic reflex, and otoacoustic emission. The occurrence of CHL in NS has been reported and is possibly due to the presence of remaining mesenchymal tissue in the middle ear and mastoid (9,11). 

In ABR, despite having good morphology, the results were atypical. That is, with the presence of CHL, the absolute latency of wave V was normal but the IPL was delayed (except for waves III-V). Typically, delayed ABR absolute latencies and normal IPLs are expected in CHL cases. For instance, in a study conducted by Matas et al. (12), the majority of CHL subjects revealed the prolongation of absolute latencies of waves I, III and V and normal IPLs. This disagreement is perhaps due to a stimulus issue. Recall that click stimuli were used to record the ABR and the ABR results represent high frequency information. As shown in the audiogram (Fig. 3), at high frequencies, the hearing level was generally within the normal range (with a slight loss at 4 kHz in the left ear). In relation to this, a CHL that occurs at low frequencies might not affect the ABR waveforms with click stimulation (13). 

The near-normal IPLs of I-V is contributed by the slight delayed IPLs of I-II and even more prolonged IPLs of II-III. Surprisingly, the IPL of III-V was abnormally shorter in the NS child compared to the normal subject. Herein, the unique ABR outcomes in the NS child are perhaps due to an abnormal anatomical condition of the auditory brainstem (e.g. compromised auditory nerve and/or cochlear nucleus). This is possible as head deformities are common in NS where CANS functions can also be affected. In the present report, the alternating clicks produced better outcomes than the rarefaction clicks when recording ABR. This finding is in line with the previous study (14). 

## Conclusion

Abnormalities in the brainstem (and perhaps other parts of CANS) might contribute to poor speech development in this NS child. In addition, delay in speech can be a result of a delay in the maturation of the nervous system (15). Nevertheless, further studies are warranted to systematically investigate this issue. ABR has good diagnostic values in determining the extent of structural abnormalities of the CANS in NS and other similar conditions involving head deformities. When performing ABR, alternating clicks should be included in the test protocol. ABR and other types of auditory evoked potentials (AEPs) should be used routinely when testing special populations. 
